# Effects of ultrasound-guided lumbar plexus and sacral plexus block combined with general anesthesia on the anesthetic efficacy and surgical outcomes in elderly patients undergoing intertrochanteric fracture surgery: a randomized controlled trial

**DOI:** 10.1186/s13018-023-04469-y

**Published:** 2024-03-07

**Authors:** Ji Feng, Guangyan Tang, Yunhua Shui, Jilin Xiang, Zhijun Qin

**Affiliations:** 1 Department of Anesthesiology, Sichuan Province Orthopedic Hospital, 132 West First Ring Road, Wuhou District, Chengdu City, 610041 Sichuan China; 2 Department of Radiology, Sichuan Province Orthopedic Hospital, Chengdu City, 610041 Sichuan China

**Keywords:** Ultrasound-guided lumbar plexus block, Sacral plexus block, General anesthesia, Intertrochanteric fracture

## Abstract

**Background:**

Surgery for intertrochanteric fractures in elderly patients is challenging due to the risk of severe pain and significant stress responses. We investigated the effects of a combined approach of ultrasound-guided lumbar plexus and sacral plexus block with general anesthesia on anesthetic efficacy and surgical outcomes in these patients.

**Methods:**

A randomized controlled trial was conducted involving 150 elderly patients, divided into two groups: the combined anesthesia group (receiving ultrasound-guided lumbar plexus and sacral plexus block along with general anesthesia) and the general anesthesia alone group. Outcome measures included hemodynamic parameters, postoperative pain levels (VAS scores), postoperative recovery times, and incidence of adverse reactions.

**Results:**

In the combined anesthesia group, the patients had more stable intraoperative hemodynamics, lower postoperative VAS scores at 1, 3, and 6 h, and faster recovery times (eye-opening upon command and return of respiratory function) compared to the general anesthesia group. Furthermore, the incidence of adverse reactions was significantly lower in the combined anesthesia group.

**Conclusions:**

Ultrasound-guided lumbar plexus and sacral plexus block combined with general anesthesia enhanced the anesthetic efficacy and improved surgical outcomes in elderly patients undergoing intertrochanteric fracture surgery.

## Introduction

Hip fractures, specifically intertrochanteric fractures, are a significant global health concern, especially among the elderly population [1, 2]. These fractures frequently arise from low-impact traumas, such as falls, which are prevalent among elderly adults due to a decrease in balance and muscular strength associated with aging [3]. The consequences of these fractures are significant, as they result in intense pain, impaired function, and a notable decline in the overall quality of life [4]. These fractures, in the most severe situations, can lead to a deterioration in the general health condition, causing the patients to become increasingly immobile and, as a result, experience higher mortality rates [5]. Timely and efficient management of intertrochanteric fractures is crucial in order to reduce their negative consequences. Surgical procedures, such as proximal femoral nail antirotation (PFNA), provide an opportunity for fast restoration of hip joint function and, consequently, earlier resumption of weight-bearing activities [6]. Nevertheless, the advantages of these operations are offset by the hazards linked to anesthesia and the surgery itself, especially in older individuals who frequently have diminished physiological reserves [7]. The surgical operation, together with the possible instability of the patient's blood flow, might have a significant impact on their recovery and survival due to the stress reaction it triggers [8].

In addition to the intrinsic risks associated with hip fractures and surgical interventions, the elderly population often presents with multiple comorbidities that compound the challenges of anesthesia and surgery [9]. Conditions such as cardiovascular diseases, respiratory disorders, and diabetes, prevalent in this demographic, can significantly enhance perioperative risks [10]. These comorbidities often necessitate a more cautious approach to anesthesia, with a critical need for techniques that minimize systemic impact while providing effective pain relief and maintaining hemodynamic stability [11]. The complex interplay of these factors underlines the necessity for anesthetic strategies that are not only effective in pain management but also considerate of the delicate health status of the elderly patient.

An optimal anesthetic strategy for senior patients undergoing surgery for intertrochanteric fractures should ensure superior pain relief, uphold stable blood flow, and promote swift recovery after the operation, all while minimizing adverse effects on the body as a whole. An effective approach in this context is the integration of regional and general anesthesia. Regional anesthetic approaches, such as lumbar plexus and sacral plexus block, provide more effective pain management for lower limb procedures, hence reducing the requirement for systemic opioids and their accompanying adverse effects [12, 13]. Additionally, they are linked to fewer changes in blood flow dynamics, which makes them an appealing choice for patients at high risk. The introduction of ultrasonic technology has completely transformed the field of regional anesthesia. The utilization of real-time imaging in nerve blocks has enhanced their safety, precision, and efficacy. The utilization of ultrasound guidance during lumbar plexus and sacral plexus block enables accurate positioning of the needle, hence minimizing the likelihood of nerve damage and enhancing the effectiveness of the block [14]. When used in conjunction with general anesthesia, this integrated method has the potential to offer the advantages of both regional and general anesthesia. This could lead to improved effectiveness of the anesthetic, decreased problems during the perioperative period, and enhanced surgical results [15].

Nevertheless, despite the theoretical merits of this method, there is limited information about its implementation and advantages in older patients who are undergoing surgery for intertrochanteric fractures. The objective of this study is to examine the efficacy of integrating ultrasound-guided lumbar plexus and sacral plexus block with general anesthesia in this particular scenario. It is postulated that this combined strategy may offer enhanced anesthetic effectiveness and lead to better surgical results, hence enhancing patient prognosis and quality of life. This research has the potential to make a substantial contribution to improving the strategies used to manage anesthesia for intertrochanteric fractures. It will provide valuable insights into a clinically important area.

## Materials and methods

### Study design

**Fig. 1:**
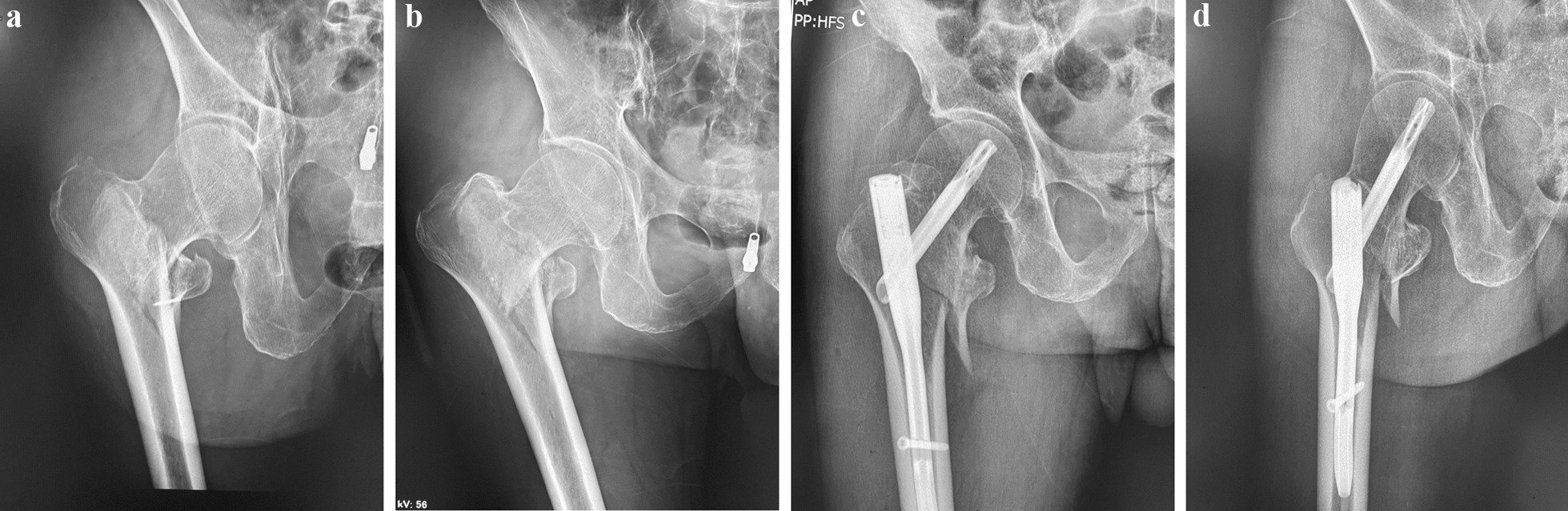
**a** Anteroposterior view of the right hip. Right intertrochanteric fracture with comminution and displacement of the lesser trochanter fragment (indicated by black arrow). **b** Oblique view of the right hip. Right intertrochanteric fracture with comminution and displacement of the lesser trochanter fragment (indicated by black arrow). **c** Anteroposterior view of the right hip. Post-internal fixation of the right intertrochanteric fracture. **d** Oblique view of the right hip. Post-internal fixation of the right intertrochanteric fracture

This study is a randomized controlled trial conducted in compliance with the Declaration of Helsinki. The study population included 150 elderly patients with intertrochanteric fractures, admitted to our institution between December 2020 and June 2022. Patients were included if they were above 70 years of age and had an intertrochanteric fracture confirmed by CT and X-ray (Fig. 1). Exclusion criteria included the presence of an infectious disease in the past three weeks, the presence of osseous tumors, and old fractures. The patients were randomly divided into two groups using a random number table, with 75 patients in each group—the combined group and the general anesthesia group. All participants provided informed consent and the study protocol was approved by the Ethics Committee of Sichuan province Orthopedic Hospital.

### Anesthetic techniques

General Anesthesia Group: Anesthesia was induced by intravenous administration of fentanyl (2–3 µg/kg), propofol (1.5 mg/kg), and vecuronium (0.15 mg/kg). Subsequently, a standard blind manual insertion of a size 3 or 4 laryngeal mask airway with an esophageal drainage port was carried out, and the cuff was inflated. Anesthesia was maintained with inhalation of sevoflurane, continuous intravenous infusion of propofol (2–3 mg/kg/min), and remifentanil (0.05 µg/kg/min), with intermittent boluses of vecuronium to maintain muscle relaxation.

Combined Group: In this technique, the patient was positioned in the bilateral leg traction position, optimal for both the surgical procedure and the administration of anesthesia. The Lumbar Plexus and Sacral Plexus blocks were administered with ultrasound guidance. For Lumbar Plexus block, the ultrasound probe was positioned in the posterior lumbar area to visualize the lumbar plexus, with the needle inserted using an in-plane technique. The Sacral Plexus block was performed by identifying the sacral hiatus, with the needle inserted under ultrasound guidance toward the sacral canal. The advantage of this approach lies in its comprehensive analgesic coverage for the entire surgical area, which is superior to other nerve blocks such as femoral nerve and lateral femoral cutaneous nerve block, ilioinguinal block, or periarticular nerve block. Furthermore, the sacral plexus block helps to alleviate the stress responses due to leg traction during surgery. Simultaneously, general anesthesia was induced and maintained at a reduced dosage due to the nerve blocks. A balanced mix of opioids and inhalational agents was used, with dosages titrated according to the patient's physiological response to ensure patient comfort and hemodynamic stability. The local anesthetic used was 0.2% Ropivacaine. The use of regional blocks allowed for a reduction in the dosage and concentration of local and general anesthetics, reducing potential systemic effects.

### Outcome measures

Our study focused on several key outcome measures to gauge the impact of the two anesthesia techniques. We monitored the hemodynamic response to surgical stress through Mean Arterial Pressure (MAP), Systolic Blood Pressure (SBP), and Heart Rate (HR) at different surgical stages. Postoperative pain was assessed using the Visual Analog Scale (VAS) at one hour, three hours, and six hours post-surgery. We also recorded the time to eye-opening and the time to recovery of respiratory function to evaluate postoperative recovery. Additionally, we tracked the incidence of adverse events to highlight any potential risks associated with each anesthesia method. These comprehensive measures enabled us to understand the varying effects of the two techniques on patient well-being and recovery.

### Statistical analysis

The statistical analysis for this study was performed using SPSS version 22.0. Data were first tested for normality. The continuous variables, which followed a normal distribution, were expressed as mean ± standard deviation (SD) and compared between groups using the independent sample *t*-test. Non-normally distributed continuous variables were analyzed using the Mann–Whitney U test and reported as median with interquartile range (IQR). Categorical variables were analyzed using the chi-square test or Fisher's exact test, as appropriate, and reported as frequencies and percentages. Baseline characteristics of the two groups were compared to ensure there was no significant difference that could influence the outcomes. Changes in the hemodynamic parameters, like MAP, SBP, and HR, were analyzed using repeated measures ANOVA to evaluate intra-group and inter-group differences over different time points. The postoperative pain scores measured by VAS at different time intervals were analyzed using the two-way repeated measures ANOVA to see if there was any significant difference in pain relief offered by the two anesthetic techniques over time. The recovery times were analyzed using the independent sample *t*-test or Mann–Whitney U test based on the data distribution. For categorical outcomes, like the incidence of adverse events, chi-square or Fisher's exact test was used, as appropriate. All tests were two-tailed, and a *P*-value of less than 0.05 was considered to indicate statistical significance.

## Results

Before the intervention, our analysis confirmed that there was no statistically significant difference between the two groups in terms of baseline characteristics. Both the combined group and the general anesthesia group showed similar demographics, health status, and pre-operative clinical measurements (*P* > 0.05), ensuring a comparable starting point for evaluating the effects of the respective anesthesia approaches.

### Stress responses comparison between groups

Before anesthesia, there were no significant differences between the combined group and the general anesthesia group regarding Mean Arterial Pressure (MAP), Systolic Blood Pressure (SBP), and Heart Rate (HR) (*P* > 0.05). However, during critical surgical moments—specifically at the instant of skin incision, 30 min into the surgery, and immediately post-surgery—the combined group exhibited statistically significant higher levels of MAP, SBP, and HR when compared to the general anesthesia group (*P* < 0.05). This observation indicates that while the combined group experienced heightened physiological stress responses at these critical moments, they still maintained relatively stable hemodynamics throughout the surgery. This contrasts with the general anesthesia group, which showed a marked decrease in these parameters, potentially indicating a less stable hemodynamic profile during these crucial surgical phases. The specifics of these comparisons are presented in Table 1.

**Table 1:** Comparison of stress responses between combined anesthesia group and general anesthesia group

Parameter	Group	Cases	Pre-anesthesia	Instant incision	30 min into surgery	Post-surgery	*t* value	*P* value
MAP (mmHg)	Combined	75	86.81 ± 8.14	82.88 ± 8.25	84.05 ± 9.07	86.69 ± 9.13	0.52	0.61
	General anesthesia	75	87.76 ± 8.57	77.21 ± 7.81	78.93 ± 7.21	81.43 ± 7.89	4.012	< 0.001
SBP (mmHg)	Combined	75	133.70 ± 12.56	120.13 ± 13.00	116.47 ± 11.53	120.94 ± 11.96	1.024	0.323
	General anesthesia	75	135.55 ± 12.96	102.86 ± 10.97	107.88 ± 11.25	111.57 ± 13.48	9.018	< 0.001
HR (bpm)	Combined	75	80.33 ± 8.53	68.77 ± 7.88	70.89 ± 8.34	71.93 ± 8.00	0.531	0.596
	General anesthesia	75	79.93 ± 7.97	61.52 ± 7.15	64.06 ± 7.40	66.86 ± 7.48	6.255	< 0.001

MAP refers to Mean Arterial Pressure, SBP refers to Systolic Blood Pressure, HR refers to Heart Rate; 1 mmHg = 0.133 kPa

### Comparison of visual analog scale scores between groups

There was no significant difference in preoperative VAS scores between the two groups (*P* > 0.05). However, at 1 h, 3 h, and 6 h postoperatively, the combined group showed significantly lower VAS scores compared to the general anesthesia group (*P* < 0.05). This indicates that the patients in the combined group experienced lower levels of pain postoperatively, which can be attributed to the effective analgesic coverage provided by the lumbar plexus and sacral plexus block. Table 2 illustrates the detailed comparison of the VAS scores between the groups.

**Table 2:** Comparison of visual analog scale (VAS) scores between combined anesthesia group and general anesthesia group

Group	Sample Size	Preoperative	Postoperative 1 h	Postoperative 3 h	Postoperative 6 h
Combined anesthesia	75	4.19 ± 0.67	1.27 ± 0.26	1.96 ± 0.30	0.93 ± 0.22
General anesthesia	75	4.60 ± 0.79	2.15 ± 0.34	2.83 ± 0.37	1.48 ± 0.27
Difference (*t*-value)		0.991	15.420	12.448	11.264
Significance (*P*-value)		0.320	< 0.001	< 0.001	< 0.001

VAS scores are utilized as a method for visual Analog scoring

### Comparison of postoperative recovery between groups

The combined group demonstrated a significantly quicker recovery in terms of eye-opening upon verbal command and respiratory function compared to the general anesthesia group (*P* < 0.05). These findings, presented in Table 3, imply that the combined approach facilitates faster postoperative cognitive and physiological recovery, potentially owing to the reduced usage of general anesthetics.

**Table 3:** Comparison of postoperative recovery between combined anesthesia group and general anesthesia group

Groups	Sample size	Time to eye opening (min)	Time to respiratory function recovery (min)	*t*-value	*P*-value
Combined anesthesia	75	21.00 ± 3.70	14.00 ± 3.10	10.350	< 0.001
General anesthesia	75	28.00 ± 4.30	19.00 ± 3.60	9.250	< 0.001

### Comparison of adverse reaction rates between groups

The incidence of adverse reactions in the combined group was significantly lower than in the general anesthesia group (*P* < 0.05). The results, detailed in Table 4, suggest that the combined approach may offer a superior safety profile, likely due to the lower amounts of general anesthetics required, thus minimizing the potential for associated side effects and complications.

**Table 4:** Comparative analysis of postoperative complications between combined and general anesthesia groups

Group	*N*	Nausea [% (*n*)]	Respiratory depression [% (*n*)]	Bradycardia [% (*n*)]	Vomiting [% (*n*)]	Total [% (*n*)]
Combined anesthesia	75	1.33 (1)	1.33 (1)	0.00 (0)	1.33 (1)	4.00 (3)
General anesthesia	75	5.33 (4)	4.00 (3)	2.67 (2)	4.00 (3)	16.00 (12)

## Discussion

Managing intertrochanteric fractures in elderly individuals poses a substantial difficulty in orthopedic surgery due to their vulnerability and deteriorated physical condition. Choosing PFNA has been regarded as a primary therapy approach for these fractures [2]. An optimal anesthetic method is crucial not just for managing pain but also for minimizing complications and deaths during surgery. The selection and execution of a proficient anesthetic plan is a crucial element in the management of patients [16]. Recently, laryngeal mask airway (LMA) general anesthesia has been used to mitigate the effects of surgical stress on the body [17]. Considering the diminished physiological capacity commonly observed in senior patients, the level of anesthesia needed for LMA (Laryngeal Mask Airway) may result in an inability to tolerate it, so compromising the effectiveness of the anesthetic [18]. Hence, it is crucial to implement an anesthetic protocol that achieves a harmonious equilibrium between efficient pain management and minimal physiological repercussions in this specific population [19, 20].

The findings of our study highlight the distinct advantages of combining regional anesthesia (RA) with LMA general anesthesia (GA) in comparison to standalone LMA GA [21]. The integration of RA into the anesthesia protocol, guided by ultrasound, directly contributed to the observed clinical outcomes in our study. Specifically, the precision in administering RA through ultrasound guidance not only reduced the risk of vascular or nerve injury but also ensured an effective anesthetic block. This precise administration resulted in an optimal balance between RA and GA, leading to enhanced hemodynamic stability and reduced pain perception, as evidenced by our study results. Furthermore, the combination of RA and GA, as opposed to standalone LMA GA, allowed for a more comprehensive anesthetic effect. The regional block, effectively executed with ultrasound guidance, minimized the need for higher doses of GA, which in turn reduced the likelihood of adverse reactions and facilitated quicker postoperative recovery. This multimodal approach effectively addresses the limitations of each anesthetic method when used alone, providing a synergistic effect that enhances overall patient outcomes [22, 23].

In our study, we primarily focused on the combined use of lumbar plexus and sacral plexus blocks with general anesthesia. However, it is noteworthy to consider other regional blocks that do not target motor components of nerves. As suggested in Pascarella et al. study [24], such approaches may offer distinct advantages, particularly in preserving motor function while providing effective analgesia. The incorporation of these techniques could further enhance patient outcomes by minimizing motor blockade, which is especially relevant in surgeries requiring postoperative mobility assessments. Safety in performing nerve blocks is a critical aspect of regional anesthesia. As referenced in Pascarella et al. study [25], the implementation of ultrasound-guided techniques, as utilized in our study, significantly reduces the risk of complications such as vascular puncture or nerve damage. This aligns with our findings, where the precise administration of the blocks contributed to lower incidence rates of adverse reactions. Our study underscores the importance of combining safety with efficacy in anesthesia, suggesting that the multimodal approach not only enhances pain management but also aligns with safety guidelines in regional anesthesia.

An intriguing aspect of our study was the nuanced understanding of the hemodynamic responses in the combined anesthesia group. Despite the initial higher levels of MAP, SBP, and HR observed in the combined group during critical surgical moments, these responses do not necessarily imply a failure in suppressing early stimulus transmission. Instead, these responses might reflect a more controlled and gradual adjustment of the body's physiological response to surgical stress, as opposed to a dramatic decline seen in the general anesthesia group. The combined approach, incorporating regional blocks, potentially modulated the sensory input from the surgical site, leading to a more stable overall hemodynamic response, even though some increase in these parameters was noted. This contrasts with the general anesthesia group, where the decline in MAP, SBP, and HR could indicate a more abrupt and possibly less controlled response to surgical stimuli. Our findings suggest that the combined anesthesia approach, while not completely eliminating the stress response to surgery, may modulate it more effectively, leading to a more stable hemodynamic profile throughout the procedure. This is supported by the statistically significant differences observed in MAP, SBP, and HR levels when comparing the combined group with the general anesthesia group at various time points during the surgery (*P* < 0.05). Our study further revealed that postoperative pain, as measured by VAS scores, was significantly lower in the combined group at 1 h, 3 h, and 6 h post-surgery. Recovery times for command-following eye-opening and respiratory function were reduced, and the incidence of adverse reactions was notably lower in the combined group (*P* < 0.05). These findings underscore the potential benefits of the combined approach in enhancing postoperative analgesia, expediting recovery, and minimizing adverse events.

Additional findings from our investigation have uncovered a significant characteristic of the integrated methodology. This approach offers total control over surgical discomfort and procedure response by efficiently suppressing both pain and motor functions in the lower leg. This is especially important because intertrochanteric fractures primarily affect the lower extremities, and the patients' ability to tolerate invasive operations may be significantly reduced due to their old age [26]. When utilized alongside LMA general anesthesia, the combination technique not only amplifies the anesthetic's quality but also enhances the outcomes of sedation. The diminished motor activity facilitates a more seamless surgical technique, while the effective pain control enhances the patient's comfort during the surgery. The balance reached between general and regional anesthesia ensures that the patient is adequately sedated, reducing the danger of intraoperative awareness, while simultaneously ensuring that the anesthetic is dispersed enough to provide surgical comfort [27]. The ultimate outcome is an ideal anesthetic condition that promotes the surgical operation while placing patient comfort and safety as the top priority.

This also has ramifications for the quantity and potency of anesthetic drugs employed. Due to its efficacy, the combined method has the potential to decrease the number of anesthetics needed to maintain the surgical level of anesthesia. This has a positive impact on mitigating the negative consequences associated with anesthetic medicines and reduces the overall physiological burden on the patient [28]. Decreasing the number of anesthetics used is also financially beneficial, resulting in lower procedure costs, which is an important factor in the present healthcare landscape. Furthermore, the combination strategy not only improves anesthetic and sedation outcomes during the surgery period, but also has a lasting impact beyond that time. By optimizing pain control and providing effective sedation, it establishes a favorable atmosphere for the postoperative healing phase [15, 29]. Patients who have reduced pain levels and a more positive experience during surgery are more inclined to express satisfaction with their surgical experience and demonstrate greater adherence to postoperative instructions, so enhancing overall surgical results.

While our study provides valuable insights into the combined approach of ultrasound-guided lumbar plexus and sacral plexus block with general anesthesia, it is essential to acknowledge certain limitations. The study, based on a statistically sufficient yet not large enough sample size, was conducted at a single medical center, which potentially limits the generalizability of the findings due to varied clinical practices and patient populations across different institutions. Although our focus on immediate and short-term postoperative outcomes provided useful data, the long-term impact of this combined anesthesia technique on patient recovery, rehabilitation, and quality of life remains unexplored. Moreover, potential observer biases due to the subjective assessment of several outcome measures, such as the VAS pain scores, may have influenced the results. Addressing these limitations in future research through increased sample sizes, multicenter collaboration, long-term follow-up, and the use of double blinding could yield more comprehensive evidence on the benefits and potential drawbacks of the combined anesthesia approach. Additionally, the absence of detailed opiate usage data affects the analysis of postoperative pain levels and potentially confounds the evaluation of anesthetic effectiveness.

In light of the suggestions for more invasive hemodynamic monitoring, we acknowledge the potential benefits this could bring to our research. Advanced hemodynamic monitoring techniques, such as arterial line placement or continuous cardiac output monitoring, could significantly enhance the precision of our hemodynamic data. These methods would allow for real-time, beat-to-beat analysis of cardiovascular responses, providing a more granular and dynamic understanding of how different anesthesia techniques affect hemodynamic stability. Incorporating these monitoring tools in future studies could help delineate the subtle physiological changes that occur during and after surgery, particularly in the elderly population who may have varying degrees of cardiovascular resilience. Such detailed hemodynamic assessment would be invaluable in tailoring anesthesia techniques to optimize patient outcomes, especially in those with pre-existing cardiovascular conditions.

## Conclusions

In conclusion, the use of ultrasound-guided lumbar plexus and sacral plexus block combined with LMA general anesthesia can yield significant benefits in elderly patients undergoing surgery for intertrochanteric fractures. This innovative approach mitigates stress responses and pain, facilitates faster recovery, and reduces the incidence of adverse reactions. The combined anesthesia method fosters a more conducive surgical environment, reducing the physiological burden on patients, enhancing surgical outcomes, and potentially boosting patient satisfaction. Future research should focus on exploring long-term benefits and potential complications of this combined approach in a larger, more diverse patient population, establishing its role as a standard of care in managing intertrochanteric fractures in elderly patients.

## Data Availability

Due to the confidentiality agreement of Sichuan Province Orthopedic Hospital and the proprietary data owned by Sichuan Province Orthopedic Hospital, the data sets generated and analyzed during this study are not public, but under reasonable requirements, the correspondence author can provide.
